# Risk of Pneumonitis Associated With Immune Checkpoint Inhibitors in Melanoma: A Systematic Review and Network Meta-Analysis

**DOI:** 10.3389/fonc.2021.651553

**Published:** 2021-10-21

**Authors:** You-Meng Sun, Wei Li, Zhi-Yu Chen, Ying Wang

**Affiliations:** ^1^ The First Affiliated Hospital, Zhejiang University School of Medicine, Hangzhou, China; ^2^ School of Statistics and Mathematics, Zhejiang Gongshang University, Hangzhou, China

**Keywords:** immune checkpoint inhibitors, network meta-analysis, melanoma, immune-related pneumonitis, systematic review

## Abstract

**Background:**

Immune checkpoint inhibitors (ICIs) have dramatically altered the treatment landscape for patients with melanoma. However, their use also generates unique immune-related adverse effects (irAEs). We performed a systematic review and network meta‐analysis to compare the risk of pneumonitis associated with ICIs for patients with advanced or metastatic melanoma.

**Methods:**

Phase II/III randomized clinical trials (RCTs) with ICIs were identified through comprehensive searches of multiple databases. An NMA was conducted to compare the risk of pneumonitis associated with ICIs and all‐grade (grade 1‐5) and high‐grade (grade 3‐5) immune‐related pneumonitis (IRP) were estimated by odds ratios (ORs).

**Results:**

A total of 10 randomized clinical trials involving 5,335 patients were enrolled in this study. Conventional chemotherapy was associated with a lower risk of grade 1–5 IRP compared with ICIs monotherapy (OR, 0.14, 95% CI, 0.03 to 0.73) and dual ICIs combination (OR, 0.03, 95% CI, 0.00 to 0.19). In addition, dual ICIs combination showed a noticeably higher risk than ICI monotherapy (OR, 4.45, 95% CI, 2.14 to 9.25) of grade 1–5 IRP. No significant difference in grade 1–5 IRP was observed between cytotoxic T lymphocyte-associated protein 4 (CTLA-4) and programmed cell death protein 1 (PD-1) inhibitors. As to grade 3‐5 IRP, no statistically significant difference was found among different ICIs-based regimens.

**Conclusion:**

These findings revealed that ICIs could increase the risk of all-grade pneumonitis for patients with advanced melanoma, compared with conventional chemotherapy. Dual ICIs combination could further increase the risk of all-grade pneumonitis than ICIs monotherapy. There was no significant difference in the risk of pneumonia between CTLA-4 and PD-1 inhibitors.

## Introduction

Melanoma is an aggressive type of skin cancer that arises from uncontrolled proliferation of melanocytes. The prevalence of melanoma has been rising steadily over the last several decades, with its incidence estimated to be increasing by 3–7% annually worldwide (1). While it represents < 5% of all cutaneous malignancies, melanoma is the major cause of death from skin cancer (2–4). If melanoma is diagnosed at early stage (stages I and II), resection of the lesion is associated with favorable survival rates. However, when diagnosed at late stage (stages III and IV), the 3-year survival rate of melanoma was reported to be 12.2% with dacarbazine chemotherapy from a multinational, randomized controlled trial (RCT) conducted from 2006 to 2008 (5).

Immune checkpoint targeted therapies including cytotoxic T lymphocyte-associated protein 4 (CTLA-4), programmed cell death protein 1 (PD-1) and programmed cell death ligand 1 (PD-L1) inhibitors have been a major breakthrough in the cancer treatment over the past decade. Immune checkpoint inhibitors (ICIs) alone as well as in combination with chemotherapy have reshaped the landscape of treatments in melanoma. Emerging evidence has demonstrated the prolonged survival with the ICIs compared with the conventional chemotherapy in patients with advanced melanoma (5–8). However, ICIs could disrupt normal mechanisms of immune regulation and lead to immune‐related adverse events (irAEs) (9). One particularly worrisome irAE is the development of immune-related pneumonitis (IRP), which typically presents with dry cough, progressive dyspnea and hypoxia along with pulmonary infiltrates on chest imaging (10).

Awareness of the risk of IRP associated with different ICIs would aid in the appropriate utilization of ICIs in clinical practice and essential monitoring of patients with ICIs treatment. Thus, we conducted a network meta-analysis (NMA) using all available data from RCTs to determine the relative risk of IRP in regards to various regimens.

## Methods

### Study Eligibility and Identification

A systematic literature search of PubMed, Embase, Cochrane Central Register of Controlled Trials was performed independently by two investigators to identify eligible RCTs in which at least one treatment arm included Food and Drug Administration (FDA) approved immune checkpoint inhibitors in patients with advanced melanoma up to October 31, 2020. The language was limited to English. The comprehensive PubMed search strategy was provided in **Supplementary Table S1**. To identify unpublished studies, the US National Institutes of Health trials register (http://clinicaltrial.gov) and conference abstracts from the American Society of Clinical Oncology (ASCO) and the European Society for Medical Oncology (ESMO) were also searched. The inclusion criteria were (1) head-to-head phase II/III RCTs which enrolled patient with pathologically confirmed advanced or metastatic melanoma; (2) patients received ICI treatment (at least one treatment arm); (3) reported the incidence of both 1–5 grade and grade 3–5 IRP. The exclusion criteria were (1) letters, reviews, unfinished studies, duplicate reports, or conference reports; (2) studies with insufficient data; (3) trials without a control arm; (4) RCTs in phase I.

### Data Extraction and Quality Assessment

The following information was extracted from eligible studies by two investigators independently: first author, year of publication, study name, National Clinical Trial (NCT) number, trial phase, treatment arms, the number of patients in total, number of patients per treatment arm, incidence of 1–5 grade and grade 3–5 IRP. Two investigators independently assessed the quality of the RCTs by using Cochrane risk assessment tool, and resolved the discrepancies through discussion and consult with a third one.

### Outcome Measures

The primary outcome of interest was all‐grade (grade 1‐5) pneumonitis. Secondary outcome was high‐grade (grade 3‐5) pneumonitis based on the National Cancer Institute Common Terminology Criteria for Adverse Events version 4.0 (11).

### Statistical Analysis

Odds ratios (ORs) and their 95% confidence intervals (CIs) were used as summary statistics to estimate treatment effects. The heterogeneity among studies was assessed using the chi-squared (χ^2^) and I-squared (I^2^) tests. We assessed the magnitude of the heterogeneity between the included publications by constructing a visual forest plot. A p value greater than 0.10 or an I^2^ value greater than 50% indicated substantial heterogeneity, and a random-effects model was used; otherwise, a fixed-effects model was used.

Inconsistency appraisal was achieved *via* two steps. First, we made a general comparison between the consistency model and the inconsistency model, calculating for inconsistency factors (IF), standard error of inconsistency factors (seIF) and p value (p). If the 95% CI of IF contained ‘0’ and the p value was greater than ‘0.05’, it was considered that direct evidence and indirect evidence were completely consistent, and there was no inconsistency. Second, node-splitting models were adopted to identify any inconsistencies. Each node of the network meta-analysis was analyzed by comparing the difference between direct comparison and indirect comparison. Significant inconsistency was defined as a p value less than 0.05. Overall, if there were no inconsistencies in the evidence, a consistency model was used to assess the relative effect of the included treatments; otherwise, an inconsistency model would be used.

The probability of treatment ranking was based on the surface under the cumulative ranking curve (SUCRA), and a higher SUCRA score was associated with a higher risk of IRP. A ‘comparison-adjusted’ funnel plot was used to assess publication bias within a network of interventions.

All statistical tests were two sided and used a significance level of p < 0.05. And we used STATA (version 15.1) for all statistical analyses.

## Results

### Study Selection

**Figure 1:**
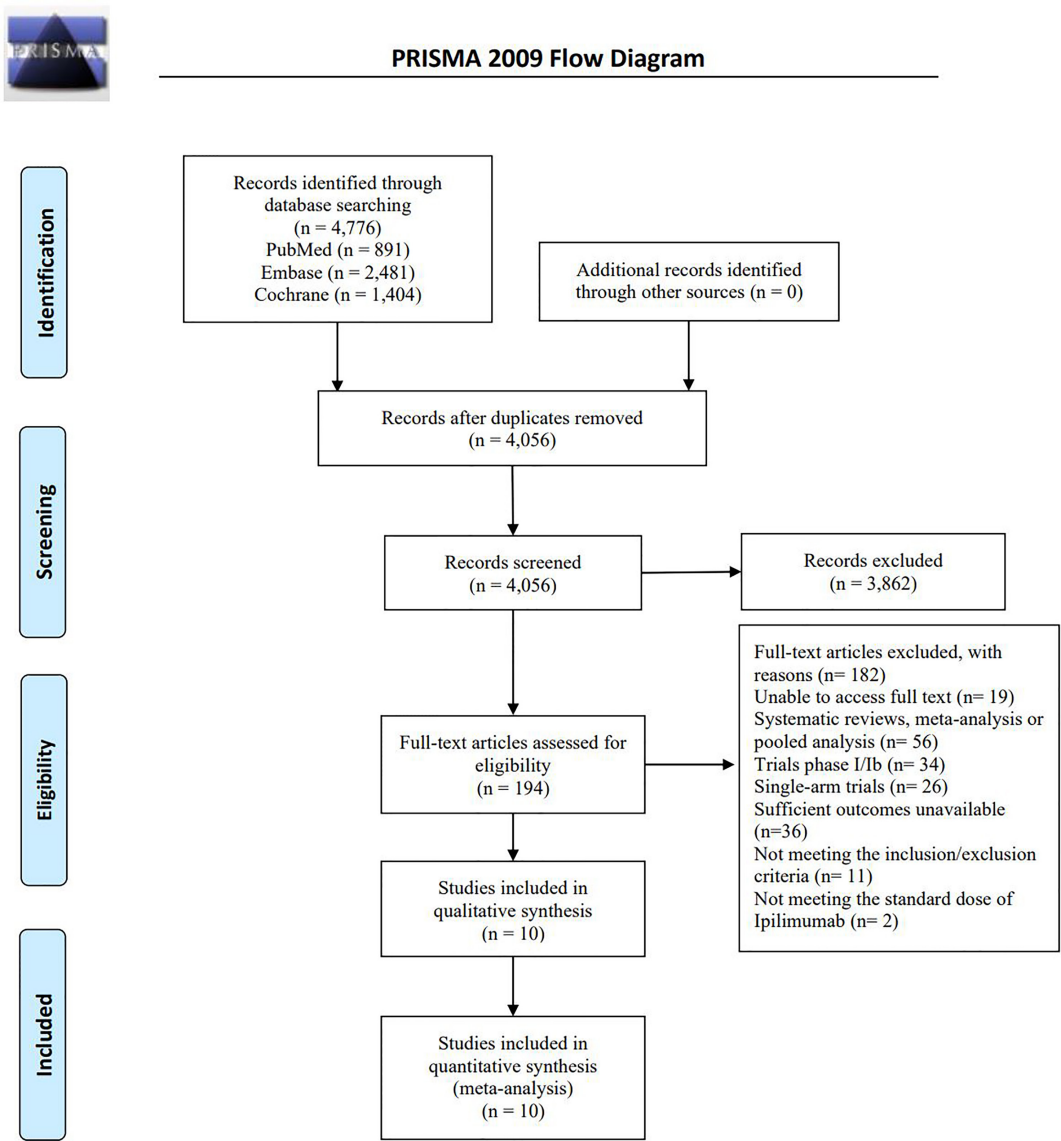
Flowchart of study search and selection.

A total of 4,776 records were initially retrieved from PubMed, Embase, Cochrane Library up to October 31, 2020. Then, 4,056 records remained after removal of the duplicates. Screening of the titles and abstracts resulted in the exclusion of 3,862 records. After full-text reading, 182 records were excluded: unable to access full text (n=19); systematic reviews, meta-analysis or pooled analysis (n=56); trials phase I/Ib (n=34); single-arm trials (n=26); sufficient outcomes unavailable (n=36); not meeting the inclusion criteria (n=11); not meeting the standard dose of ipilimumab (n=2). In total, 10 eligible randomized trials (6, 7, 12–19) were included in this study. The PRISMA flow diagram of study selection is shown as follows (**Figure 1**).

The Cochrane tool for risk of bias was used to measure the quality of each study. The detailed assessment results were shown in **Supplementary Figure S1**. No obvious publication bias was observed in this NMA; the funnel plots were roughly symmetrical and near the zero line (**Supplementary Figure S2–S5**).

### Patient Characteristics and Treatment Group Description

There were 6 phase III trials, 4 phase II trials (**Table 1**). Among the 10 eligible RCTs, 8 studies were two-arm trials and 2 studies were three-arm trials. There were 22 treatment arms in total and the most common treatment arm was ICIs monotherapy (n=13, 59.1%), followed by dual ICIs combination (n=4, 18.2%), chemotherapy (n=3, 13.6%) and placebo (n=2, 9.1%). The ICIs used for the melanoma treatment included ipilimumab (CTLA-4), nivolumab (PD-1) and pembrolizumab (PD-1) while chemotherapy regimens included carboplatin, dacarbazine, and/or paclitaxel. A total of 5,335 patients were included in this study and the sample size ranged from 60 to 1,011.

**Table 1:** Characteristics of included studies.

Authors, Year	Study Name	NCT Number	Phase	Malignancy	Treatment arms	Patient ‘number	Pneumonitis	Pneumonitis
							(Any Grade, n)	(Grade3-5, n)
Robert et al. (7)	Checkmate 066	NCT01721772	III	Melanoma	Nivolumab	206	3	0
					Dacarbazine	205	0	0
Postow et al. (11)	Checkmate 069	NCT01927419	II	Melanoma	Nivolumab + Ipilimumab	94	10	3
					Ipilimumab	46	2	1
Larkin et al. (12)	Checkmate 037	NCT01721746	III	Melanoma	Nivolumab	268	7	0
					ICC	102	0	0
Hamid et al. (6)	KEYNOTE-002	NCT01704287	II	Melanoma	Pembrolizumab	357	9	5
					Chemotherapy	171	0	0
Long et al. (13)	CA209-170	NCT02374242	II	Melanoma	Nivolumab + Ipilimumab	35	5	1
					Nivolumab	25	1	0
Robert et al. (14)	KEYNOTE-006	NCT01866319	III	Melanoma	Combined pembrolizumab groups	555	8	4
					Ipilimumab	256	2	1
Larkin et al. (15)	Checkmate 067	NCT01844505	III	Melanoma	Nivolumab + Ipilimumab	313	23	3
					Nivolumab	313	5	1
					Ipilimumab	311	5	1
Eggermont et al. (16)	KEYNOTE-054	NCT02362594	III	Melanoma	Pembrolizumab	509	17	4
					Placebo	502	3	0
Zimmer et al. (17)	IMMUNED	NCT02523313	II	Melanoma	Nivolumab + Ipilimumab	55	8	0
					Nivolumab	56	0	0
					Placebo	51	0	0
Ascierto et al. (18)	Checkmate 238	NCT02388906	III	Melanoma	Nivolumab	452	1	1
					Ipilimumab	453	0	0

ICC: dacarbazine 1000 mg/m² every 3 weeks or paclitaxel 175 mg/m² combined with carboplatin area under the curve 6 every 3 weeks; Combined pembrolizumab groups: pembrolizumab (at a dose of 10 mg per kilogram of body weight) every 2 weeks or every 3 weeks.

The rate of IRP in different treatment regimens was compared among four groups including chemotherapy, ICIs monotherapy, dual ICIs combination and placebo. Furthermore, we subdivided the four treatment groups into six subgroups based on the different types of ICIs: chemotherapy, ipilimumab, nivolumab, nivolumab+ipilimumab, pembrolizumab and placebo.

### NMA for IRP Based on Four Treatment Groups

**Figure 2:**
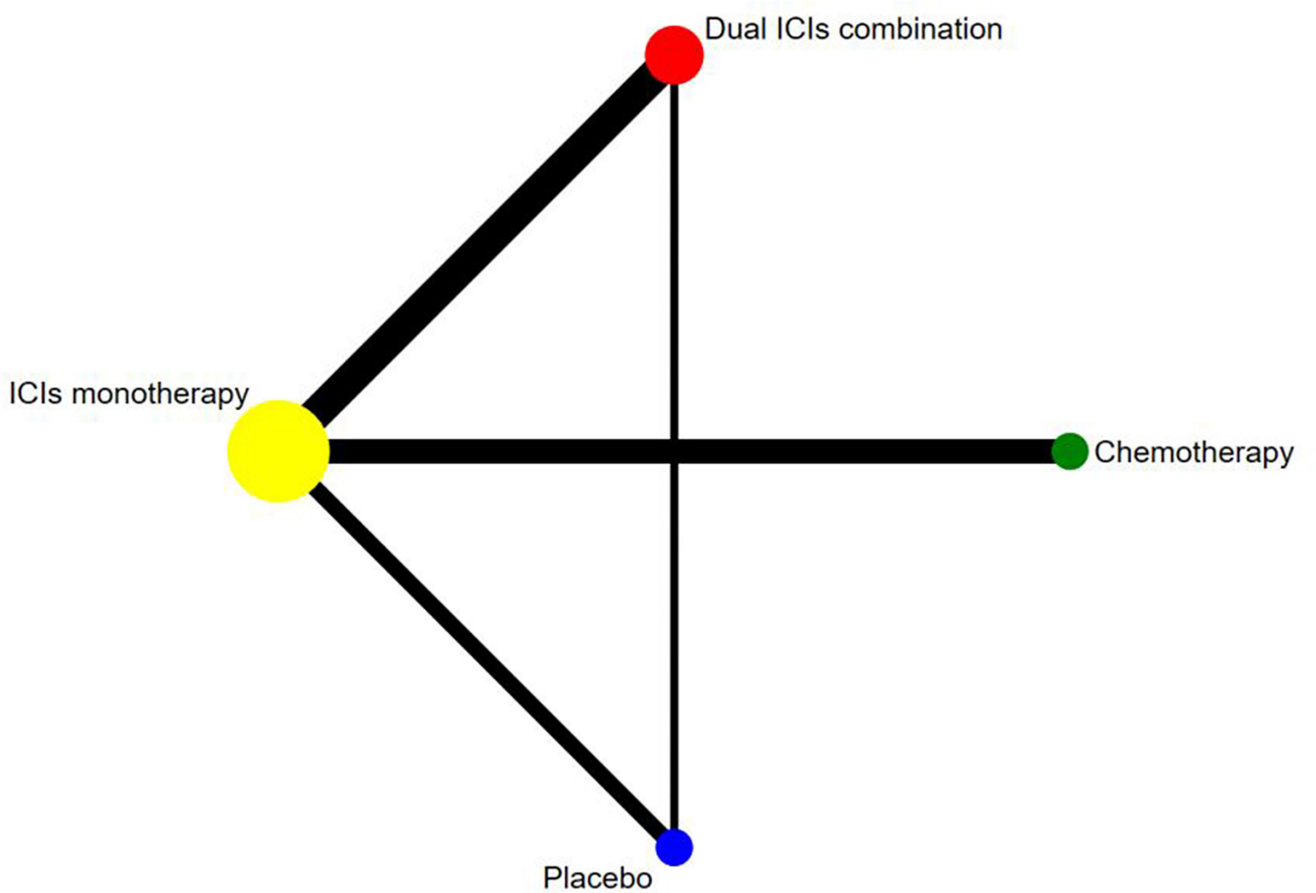
Network established for comparisons based on four treatment groups. Circular nodes indicate treatment regimens. The node size corresponds with the total number of patients randomized to receive the treatment. Each line represents a type of head-to-head comparison. The width of the lines is proportional to the number of trials comparing the connected treatments.

According to the established NMA based on the consistency model (**Figure 2** and **Table 2**), chemotherapy had a lower risk of grade 1–5 IRP compared with dual ICIs combination (OR, 0.03, 95% CI, 0.00 to 0.19) and ICI monotherapy (OR, 0.14, 95% CI, 0.03 to 0.73). In addition, dual ICIs combination showed a noticeably higher risk than ICI monotherapy (OR, 4.45, 95% CI, 2.14 to 9.25). Compared with placebo, dual ICIs combination (OR, 23.94, 95% CI, 6.56 to 87.38) and ICI monotherapy (OR, 5.38, 95% CI, 1.72 to 16.80) were associated with a considerable higher risk of grade 1–5 IRP. As to high‐grade (grade 3‐5) IRP, no statistically significant difference was found among the four treatment groups (**Table 3**).

**Table 2:** Multiple treatment comparison for IRP based on network consistency model (for grade 1–5 IRP).

OR with 95% CI for grade 1–5 IRP			
Chemotherapy	32.53 (5.24,201.82)	7.31 (1.37,38.91)	1.36 (0.18,10.27)
**0.03 (0.00,0.19)**	Dual ICIs combination	0.22 (0.11,0.47)	0.04 (0.01,0.15)
**0.14 (0.03,0.73)**	**4.45 (2.14,9.25)**	ICIs monotherapy	0.19 (0.06,0.58)
0.74 (0.10,5.56)	**23.94 (6.56,87.38)**	**5.38 (1.72,16.80)**	Placebo

The bold values mean that there exist significant differences in the risk of IRP between two different treatment groups.

**Table 3:** Multiple treatment comparison for IRP based on network consistency model (for grade 3–5 IRP).

OR with 95% CI for grade 3–5 IRP			
Chemotherapy	3.02 (0.27,33.74)	1.73 (0.23,12.87)	0.57 (0.03,11.53)
0.33 (0.03,3.69)	Dual ICIs combination	0.57 (0.15,2.19)	0.19 (0.02,2.21)
0.58 (0.08,4.29)	1.75 (0.46,6.67)	ICIs monotherapy	0.33 (0.04,3.09)
1.75 (0.09,35.35)	5.30 (0.45,61.89)	3.03 (0.32,28.43)	Placebo

**Figure 3:**
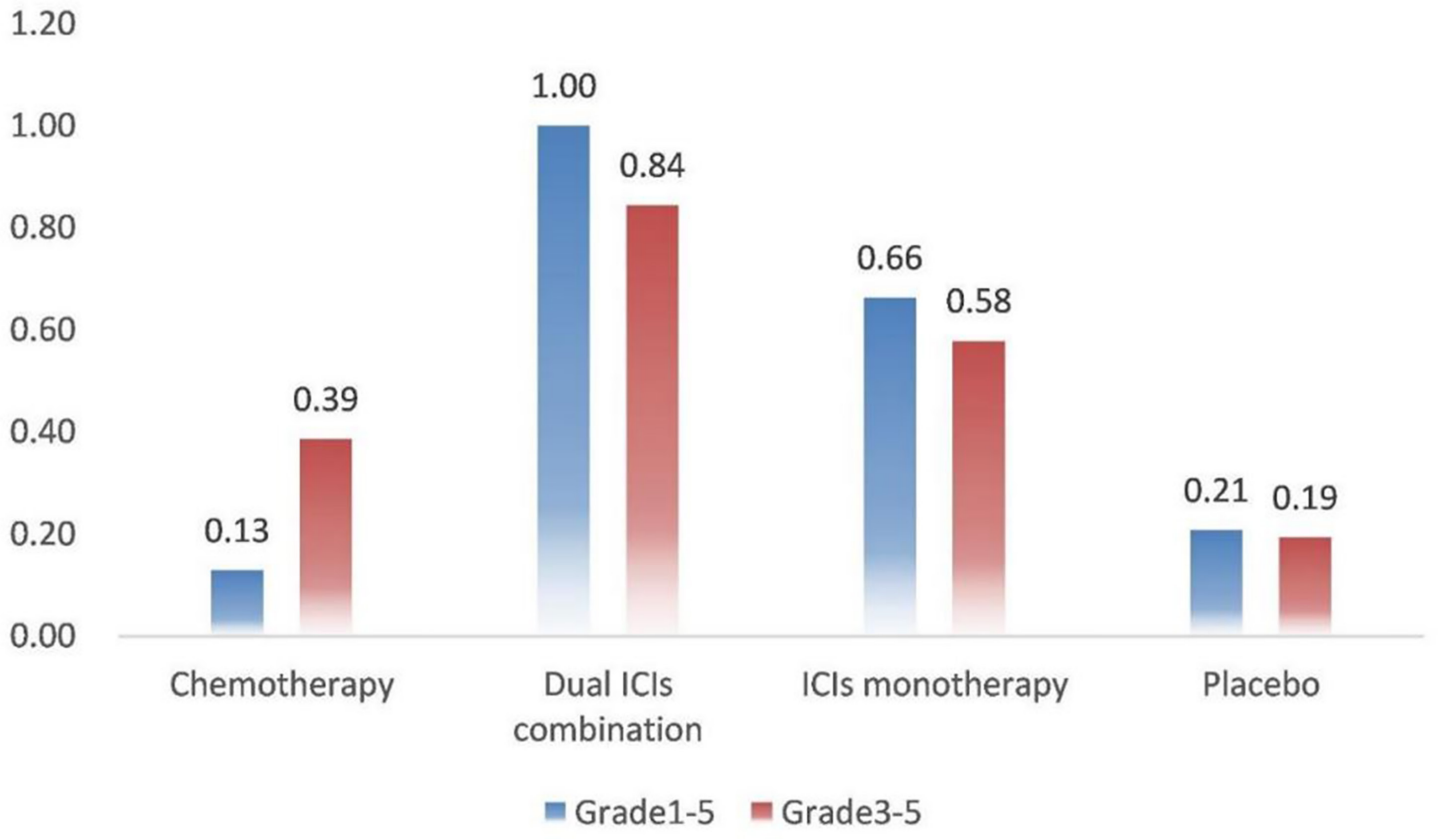
Rank probabilities with SUCRA value for IRP in four treatment groups based on the network consistency model. Higher SUCRA scores are correlated with higher risk of IRP. SUCRA, surface under the cumulative ranking curve.

SUCRA provided a ranking of the four treatment groups according to the incidence of IRP (**Figure 3**). For grade 1–5 IRP, dual ICIs combination was with the highest ranking (1.00), followed by ICI monotherapy (0.66), placebo (0.21) and chemotherapy (0.13). The ranking of grade 3–5 IRP was consistent with grade 1–5 IRP, with dual ICIs combination (0.84) ranking the highest, followed by ICI monotherapy (0.58), chemotherapy (0.39) and placebo (0.19).

### NMA for IRP by Different ICIs Based on Six Treatment Groups

To further compare the difference of IRP among various ICIs, including ipilimumab, nivolumab and pembrolizumab, another NMA for IRP by different ICIs based on six treatment groups was built (**Supplementary Figure S6** and **Table S2**). Chemotherapy was with a lower risk of grade 1–5 IRP, compared with nivolumab (OR, 0.17, 95% CI, 0.03 to 0.97), nivolumab+ ipilimumab (OR, 0.04, 95% CI, 0.01 to 0.22) and pembrolizumab (OR, 0.09, 95% CI, 0.01 to 0.64). Both ipilimumab (OR, 0.22, 95% CI, 0.10 to 0.49) and nivolumab (OR, 0.21, 95% CI, 0.09 to 0.47) were associated with a lower risk, when compared with their combination (nivolumab+ ipilimumab). The risk of 1-5 IRP was comparable among nivolumab, pembrolizumab and ipilimumab. Therefore, it could be inferred that there was no significant difference between CTLA-4 and PD-1 inhibitors. In terms of grade 3‐5 IRP, no significant difference was seen among the six treatment groups (**Supplementary Table S3**).

The ranking of grade 1–5 IRP from high to low was: nivolumab+ipilimumab (0.98), pembrolizumab (0.72), chemotherapy (0.70), nivolumab (0.51), placebo (0.18) and ipilimumab (0.00). As to grade 3‐5 IRP, the ranking was pembrolizumab (0.85), nivolumab+ipilimumab (0.68), chemotherapy (0.42), ipilimumab (0.38), nivolumab (0.38) and placebo (0.29) (**Supplementary Figure S7**).

### Heterogeneity and Inconsistency Assessment

Pairwise comparisons with heterogeneity estimates are presented in **Supplementary Figures S8–S11**. The results showed low heterogeneity in relation to the NMA results. In addition, the results of the inconsistency evaluation are presented in **Supplementary Tables S4–S7**. No significant inconsistency was observed between direct and indirect studies. The model’s overall fit was satisfactory.

## Discussion

Immune checkpoint inhibitors have now become the first-line treatment options for advanced melanoma in the US according to the National Comprehensive Cancer Network guidelines (20). ICIs are increasingly used in cancer therapy and a key challenge we have to face is the uncontrolled collateral effects on the immune system (21). Immune-related adverse events are autoimmune-toxic effects associated with ICIs used for the treatment of advanced solid tumors (22). The differences in the risk of irAEs may attribute to the different mechanisms of each agent and the combined use of ICIs (23). Ipilimumab is a cytotoxic T-lymphocyte–associated protein 4 inhibitor that increases T cell function and antitumor responses in patients with advanced melanoma, through suppressing T cell effector function following initial activation by costimulatory signals (24). Pembrolizumab and nivolumab are both PD-1 inhibitors, that reinvigorate tumor-specific exhausted T cells and promote immune-mediated elimination of tumor cells (25, 26). Theoretically, CTLA-4 inhibitors may induce a greater magnitude of T cell proliferation or reduced regulatory T cell-mediated immunosuppression, while PD-1 inhibitors may activate a relatively smaller number of T cells (27, 28). Thus, PD-1 inhibitors are often considered to be better tolerated than CTLA-4 inhibitor (29). However, in reality, the types of irAEs related to monotherapy targeting the CTLA-4 or PD-1 differ (30). One previous study revealed that rash, colitis and hypophysitis were more common with CTLA-4 inhibitors; pneumonitis, arthralgia and hypothyroidism were more frequently seen with PD-1 inhibitors (31). The biomedical explanations for the differences in irAE localization with different ICIs have not yet been fully elucidated.

In this study, we found that ICIs were associated with a higher risk of grade 1–5 IRP compared with conventional chemotherapy in melanoma patients. In addition, dual ICIs combination showed a higher risk than ICI monotherapy. It was implied that ICIs could increase the risk of IRP in melanoma patients compared with conventional chemotherapy, which was consistent with previous studies that conducted in lung cancer (32) and other solid tumors (33). When comparing the risk of IRP among three kinds of ICI monotherapy (ipilimumab, nivolumab and pembrolizumab), no significant difference was observed in this study. It could be inferred that the risk of IRP was comparable among the three kinds of ICI monotherapy and there was no significant difference between CTLA-4 and PD-1 inhibitors. It was inconsistent with the aforementioned conclusion that IRP was more common with PD-1 inhibitors (31). It is worth mentioning that there were only ten eligible randomized clinical trials enrolled in this study and most assessments of IRP risk of ICIs come from comparisons between ICI monotherapy and dual ICIs combination or chemotherapy. There were no randomized clinical trials that evaluated IRP risk involving ICI in combination with chemotherapy. It is implausible to see the higher ranking of placebo *versus* chemotherapy in terms of grade 1‐5 IRP, although we have demonstrated there were no statistically significant differences between placebo and chemotherapy. It was probably due to the limited number of eligible RCTs and relatively small sample size. In addition, no statistically significant differences were seen between the four treatment groups in the risk of high‐grade (grade 3‐5) IRP. It could mainly attribute to the low prevalence of high-grade IRP. Despite our current study provides insight in indirect comparisons, head‐to‐head comparisons among different ICI-based regimens are still lacking. More well-constructed, adequately powered randomized clinical trials should be conducted to assess the safety of the combination of ICIs and chemotherapy to enrich the evidence.

With the increasing application of ICIs in cancer treatment, there is undoubtedly a rise in the absolute burden and mortality of pneumonitis. It is important to define a tailored treatment strategy to maximize the treatment benefits and minimize immune‐related adverse events, especially serious or fatal adverse events. In the present study, we found that the combination of two ICIs was associated with a higher risk of irAEs compared with the monotherapy alone. Previous studies suggested that the incidence of pneumonitis with combination therapy may be higher and the time to onset is sooner. The median time to onset of pneumonitis was 2.7 months in patients with dual ICIs combination and 4.6 months in patients with ICI monotherapy (34). Furthermore, some research groups found that the patients who experienced irAEs had significantly better clinical outcomes (35, 36). There have been other groups that reported the safety of resuming immunotherapy after immune-related toxicity (37, 38). The Society for Immunotherapy of Cancer recommended that resuming ICI therapy remained to be an available treatment option in patients in patients with grade 2-3 pneumonitis, which has resolved completely (39). Our findings may provide important implications for better clinical practice guidance on ICI use in terms of irAEs (40, 41). For patients with preexisting autoimmune diseases, prior radiotherapy and heavy smoking history, ICI monotherapy at low dose initially is recommended to avoid irAEs, instead of ICI combination. In addition, intensive monitoring is essential for early identification and intervention.

To the best of our knowledge, this is the first systematic review and network meta-analysis which provides the most updated and comprehensive evidence of IRP for ICIs related therapeutic regimens in melanoma patients. This study had several limitations that should be acknowledged. First, no consensus diagnostic criteria of IRP are available and the identification of IRP in different studies may not be completely homogeneous. In addition, some low-grade IRP related symptoms such as cough, malaise and mild fever are not specific and likely ignored by clinicians. Therefore, the identification of IRP might not be accurate and complete, which would lead to bias for the assessment of IRP. Second, 30% of the enrolled trials in this study were open-label and may bring unconscious bias. Third, the reported incidence of IRP would increase gradually with longer follow-up and more patients receiving ICIs. IRP is an uncommon but potentially fatal toxicity that results in a high rate of treatment discontinuation and mortality in patients. It implies that IRP requires clinicians to pay close attention, and formulate corresponding prevention. Fourth, commonly accepted risk factors for IRP such as prior radiotherapy and smoking history were potential confounders for the evaluation of risk of IRP. Last, patients may receive corticosteroids when pneumonitis occurred. As is known to all, corticosteroids could suppress the immune system, reduce inflammation and relieve the development of IRP. However, how immune checkpoint inhibitor agents and corticosteroids suppress and regulate the immune system has not been clarified. There has been preliminary evidence that the use of corticosteroids for IRP did not influence the effectiveness of ICIs (42). More relevant works including fundamental researches and clinical trials are in urgent need to address this important clinical issue.

In summary, our network meta-analysis has demonstrated that ICI-based therapy is associated with a higher risk of IRP than chemotherapy in melanoma patients. Dual ICIs combination therapy has a higher risk of IRP than ICI monotherapy. There was no significant difference between CTLA-4 and PD-1 inhibitors in terms of risk of IRP. These findings may provide important implication for making individualized treatment therapy for melanoma patients.

## Data Availability Statement

The original contributions presented in the study are included in the article/**Supplementary Material**. Further inquiries can be directed to the corresponding author.

## Author Contributions

Y-MS and WL contributed equally to the work. Y-MS and WL finished the initial design and conception of the research. Y-MS, WL, and Z-YC contributed to the acquisition of data, analysis and interpretation. YW participated in drafting and revising the article. All authors contributed to the article and approved the submitted version.

## Conflict of Interest

The authors declare that the research was conducted in the absence of any commercial or financial relationships that could be construed as a potential conflict of interest.

## Publisher’s Note

All claims expressed in this article are solely those of the authors and do not necessarily represent those of their affiliated organizations, or those of the publisher, the editors and the reviewers. Any product that may be evaluated in this article, or claim that may be made by its manufacturer, is not guaranteed or endorsed by the publisher.

## Abbreviations

ICIs, immune checkpoint inhibitors; irAEs, immune-related adverse effects; RCT, randomized clinical trial; NMA, network meta-analysis; IRP, immune‐related pneumonitis; ORs, odds ratios; CTLA-4, cytotoxic T lymphocyte-associated protein 4; PD-1, programmed cell death protein 1; PD-L1, programmed cell death ligand 1; FDA, Food and Drug Administration; ASCO, American Society of Clinical Oncology; ESMO, European Society of Medical Oncology; NCT, National Clinical Trial; CIs, confidence intervals; IF, inconsistency factors; seIF, standard error of inconsistency factors; SUCRA, surface under the cumulative ranking curve.
