# Supplementary material for: Risk of Pneumonitis Associated With Immune Checkpoint Inhibitors in Melanoma: A Systematic Review and Network Meta-Analysis
Source: Front Oncol. 2021 Oct 21;11:651553. doi: 10.3389/fonc.2021.651553 (PMC8568299; doi:10.3389/fonc.2021.651553)

| **Table S1 Search Strategy** | | |
| --- | --- | --- |
| **PubMed** | | |
| **Step** | **Query** | **Items found** |
| **#1:** | **Search: "Melanoma"[MeSH Terms] OR "Melanoma"[Title/Abstract] OR "Melanomas"[Title/Abstract] OR "malignant melanoma"[Title/Abstract] OR "malignant melanomas"[Title/Abstract] OR "melanoma malignant"[Title/Abstract] OR "melanomas malignant"[Title/Abstract]** | **136545** |
| **#2:** | **Search: "Anti-programmed-death-receptor-1"[Title/Abstract] OR "programmed death ligand 1"[Title/Abstract] OR "PD-L1"[Title/Abstract] OR "programmed death 1"[Title/Abstract] OR "PD-1"[Title/Abstract] OR "anti-programmed death ligand 1"[Title/Abstract] OR "Anti-PD-L1"[Title/Abstract] OR "anti-programmed death 1"[Title/Abstract] OR "Anti-PD-1"[Title/Abstract] OR "Nivolumab"[Title/Abstract] OR "Pembrolizumab"[Title/Abstract] OR "Avelumab"[Title/Abstract] OR "Atezolizumab"[Title/Abstract] OR "Durvalumab"[Title/Abstract] OR "Cemiplimab"[Title/Abstract] OR "anti-cytotoxic t lymphocyte antigen 4"[Title/Abstract] OR "Anti-CTLA-4"[Title/Abstract] OR "Ipilimumab"[Title/Abstract] OR "Tremelimumab"[Title/Abstract] OR "Immunotherapy"[Title/Abstract] OR "immune checkpoint inhibitors"[Title/Abstract] OR "ICI"[Title/Abstract]** | **111350** |
| **#3:** | **Search: "clinical trial"[Title/Abstract] OR "controlled clinical trial"[Title/Abstract] OR "randomized controlled trial"[Title/Abstract] OR "RCT"[Title/Abstract] OR "random"[Title/Abstract] OR "randomized"[Title/Abstract]** | **898300** |
| **#4** | **#1 AND #2 AND #3** | **891** |
| **Embase** | | |
| **Step** | **Query** | **Collapse** |
| **#1** | 'melanoma'/exp | **172036** |
| **#2** | 'melanoma':ab,ti | **163179** |
| **#3** | 'melanomas':ab,ti | **26324** |
| **#4** | 'malignant melanoma':ab,ti | **33539** |
| **#5** | 'malignant melanomas':ab,ti | **4225** |
| **#6** | 'melanoma malignant':ab,ti | **131** |
| **#7** | 'melanomas malignant':ab,ti | **20** |
| **#8** | #1 OR #2 OR #3 OR #4 OR #5 OR #6 OR #7 | **210237** |
| **#9** | 'anti-programmed-death-receptor-1':ab,ti | **67** |
| **#10** | 'programmed death ligand 1':ab,ti | **5531** |
| **#11** | 'pd-l1':ab,ti | **29535** |
| **#12** | 'programmed death 1':ab,ti | **5108** |
| **#13** | 'pd-1':ab,ti | **34079** |
| **#14** | 'anti-programmed death ligand 1':ab,ti | **268** |
| **#15** | 'anti-pd-l1':ab,ti | **3964** |
| **#16** | 'anti-programmed death 1':ab,ti | **588** |
| **#17** | 'anti-pd-1':ab,ti | **9759** |
| **#18** | 'nivolumab':ab,ti | **12283** |
| **#19** | 'pembrolizumab':ab,ti | **10315** |
| **#20** | 'avelumab':ab,ti | **1118** |
| **#21** | 'atezolizumab':ab,ti | **2944** |
| **#22** | 'durvalumab':ab,ti | **1798** |
| **#23** | 'cemiplimab':ab,ti | **221** |
| **#24** | 'anti-cytotoxic t lymphocyte antigen 4':ab,ti | **176** |
| **#25** | 'anti-ctla-4':ab,ti | **3025** |
| **#26** | 'ipilimumab':ab,ti | **7528** |
| **#27** | 'tremelimumab':ab,ti | **693** |
| **#28** | 'Immunotherapy':ab,ti | **123357** |
| **#29** | 'immune checkpoint inhibitors':ab,ti | **13155** |
| **#30** | 'ici':ab,ti | **13408** |
| **#31** | #9 OR #10 OR #11 OR #12 OR #13 OR #14 OR #15 OR #16 OR #17 OR #18 OR #19 OR #20 OR #21 OR #22 OR #23 OR #24 OR #25 OR #26 OR #27 OR #28 OR #29 OR #30 | **181767** |
| **#32** | 'clinical trial':ab,ti | **223515** |
| **#33** | 'controlled clinical trial':ab,ti | **20406** |
| **#34** | 'randomized controlled trial':ab,ti | **106283** |
| **#35** | 'rct':ab,ti | **41312** |
| **#36** | 'random':ab,ti | **347794** |
| **#37** | 'randomized':ab,ti | **796062** |
| **#38** | #32 OR #33 OR #34 OR #35 OR #36 OR #37 | **1264705** |
| **#39** | #8 AND #31 AND #38 | **2481** |
| **Cochrane** | | |
| **Step** | **Search manager** | **Items found** |
| **#1** | MeSH descriptor: [Melanoma] explode all trees | **1838** |
| **#2** | (Melanoma):ti,ab,kw OR (Melanomas):ti,ab,kw OR (malignant melanoma):ti,ab,kw OR (malignant melanomas):ti,ab,kw OR (melanoma malignant):ti,ab,kw | **5525** |
| **#3** | **melanomas malignant** | **1364** |
| **#4** | **#1 OR #2 OR #3** | **5527** |
| **#5** | (Anti-programmed-death-receptor-1):ti,ab,kw OR (programmed death ligand 1):ti,ab,kw OR (PD-L1):ti,ab,kw OR (programmed death 1):ti,ab,kw OR (PD-1):ti,ab,kw | **6283** |
| **#6** | (anti programmed death ligand 1):ti,ab,kw OR (Anti-PD-L1):ti,ab,kw OR (anti programmed death 1):ti,ab,kw OR (Anti-PD-1):ti,ab,kw OR (Nivolumab):ti,ab,kw | **3181** |
| **#7** | (Pembrolizumab):ti,ab,kw OR (Avelumab):ti,ab,kw OR (Atezolizumab):ti,ab,kw OR (Durvalumab):ti,ab,kw OR (Cemiplimab):ti,ab,kw | **3197** |
| **#8** | (anti cytotoxic t lymphocyte antigen 4):ti,ab,kw OR (Anti-CTLA-4):ti,ab,kw OR (Ipilimumab):ti,ab,kw OR (Tremelimumab):ti,ab,kw OR (Immunotherapy):ti,ab,kw | **11313** |
| **#9** | (immune checkpoint inhibitors):ti,ab,kw OR (ICI):ti,ab,kw | **1528** |
| **#10** | #5 OR #6 OR #7 OR #8 OR #9 | **18357** |
| **#11** | #4 AND #11 | **1404** |

**Table S2** **Multiple treatment comparison for IRP based on network consistency model (for grade 1–5 IRP).**

| OR with 95% CI for grade 1–5 IRP | |  | |  | |  | |
| --- | --- | --- | --- | --- | --- | --- | --- |
| Chemotherapy | 6.34 (0.97,41.50) | | 5.92 (1.03,34.11) | 28.55 (4.50,180.96) | 10.93 (1.56,76.39) | | 1.87 (0.21,16.47) |
| 0.16 (0.02,1.03) | Ipil | | 0.93 (0.34,2.54) | 4.51 (2.06,9.86) | 1.72 (0.46,6.44) | | 0.29 (0.06,1.47) |
| **0.17 (0.03,0.97)** | 1.07 (0.39,2.91) | | Niv | 4.82 (2.13,10.93) | 1.85 (0.41,8.39) | | 0.32 (0.05,1.81) |
| **0.04 (0.01,0.22)** | **0.22 (0.10,0.49)** | | **0.21 (0.09,0.47)** | Niv+Ipil | 0.38 (0.09,1.58) | | 0.07 (0.01,0.34) |
| **0.09 (0.01,0.64)** | 0.58 (0.16,2.17) | | 0.54 (0.12,2.46) | 2.61 (0.63,10.81) | Pem | | 0.17 (0.05,0.54) |
| 0.54 (0.06,4.72) | 3.39 (0.68,16.93) | | 3.17 (0.55,18.18) | **15.28 (2.93,79.66)** | **5.85 (1.84,18.56)** | | Placebo |

**Table S3** **Multiple treatment comparison for IRP based on network consistency model (for grade 3–5 IRP).**

| OR with 95% CI for grade 3–5 IRP | |  | |  | |  | |
| --- | --- | --- | --- | --- | --- | --- | --- |
| Chemotherapy | 0.99 (0.09,11.31) | | 0.95 (0.10,8.96) | 1.95 (0.16,23.65) | 3.34 (0.34,32.83) | | 0.67 (0.03,13.75) |
| 1.01 (0.09,11.58) | Ipil | | 0.96 (0.19,4.95) | 1.97 (0.47,8.27) | 3.37 (0.53,21.33) | | 0.68 (0.05,9.01) |
| 1.05 (0.11,9.92) | 1.04 (0.20,5.35) | | Niv | 2.05 (0.45,9.24) | 3.51 (0.43,28.79) | | 0.70 (0.05,9.50) |
| 0.51 (0.04,6.24) | 0.51 (0.12,2.13) | | 0.49 (0.11,2.20) | Niv+Ipil | 1.71 (0.21,14.18) | | 0.34 (0.03,4.65) |
| 0.30 (0.03,2.95) | 0.30 (0.05,1.87) | | 0.28 (0.03,2.34) | 0.58 (0.07,4.83) | Pem | | 0.20 (0.02,2.15) |
| 1.50 (0.07,30.76) | 1.48 (0.11,19.70) | | 1.42 (0.11,19.20) | 2.91 (0.21,39.48) | 4.99 (0.47,53.47) | | Placebo |

**Table S4 Evaluation of inconsistency for four treatment groups**

| Outcomes | Cycle | Inconsistency Factors (IF) | seIF | CI_95 | P Value |
| --- | --- | --- | --- | --- | --- |
| Grade 1-5 IRP | Dual ICIs combination-ICIs monotherapy-Placebo | 0.077 | 1.636 | (0.00,3.28) | 0.962 |
| Grade 3-5 IRP | Dual ICIs combination-ICIs monotherapy-Placebo | 2.216 | 2.452 | (0.00,7.02) | 0.904 |

**Table S5 Results of Node-splitting analysis for the assessment of inconsistency for four treatment groups**

| Comparator | Intervention | Direct | | Indirect | | Difference | | P | Tau |
| --- | --- | --- | --- | --- | --- | --- | --- | --- | --- |
|  |  | Coef. | Std. Err. | Coef. | Std. Err. | Coef. | Std. Err. |  |  |
| **Grade 1-5 IRP** | | | | | | | | | |
| Chemotherapy | ICIs monotherapy | 1.99 | 0.85 | -1.19 | 141.25 | 3.18 | 141.25 | 0.98 | 0.00 |
| Dual ICIs combination | ICIs monotherapy | -1.52 | 0.38 | -0.62 | 3.21 | -0.89 | 3.27 | 0.79 | 0.00 |
| Dual ICIs combination | Placebo | -3.51 | 1.35 | -3.08 | 0.74 | -0.43 | 1.51 | 0.77 | 0.00 |
| ICIs monotherapy | Placebo | -1.58 | 0.60 | -3.11 | 2.28 | 1.52 | 2.36 | 0.52 | 0.00 |
| **Grade 3-5 IRP** | | | | | | | | | |
| Chemotherapy | ICIs monotherapy | 0.55 | 1.02 | -0.45 | 141.26 | 1.00 | 141.27 | 0.99 | 0.00 |
| Dual ICIs combination | ICIs monotherapy | -0.67 | 0.69 | 4.55 | 4.58 | -5.22 | 4.64 | 0.26 | 0.00 |
| Dual ICIs combination | Placebo | 0.07 | 2.01 | -2.78 | 1.61 | 2.85 | 2.57 | 0.27 | 0.00 |
| ICIs monotherapy | Placebo | -1.38 | 1.20 | 1.58 | 3.78 | -2.96 | 3.96 | 0.46 | 0.00 |

**Table S6 Evaluation of inconsistency for six treatment groups**

| Outcomes | Cycle | Inconsistency Factors (IF) | seIF | CI_95 | P Value |
| --- | --- | --- | --- | --- | --- |
| Grade 1-5 IRP | Ipil-Niv-Placebo | 1.47 | 2.77 | (0.00,6.89) | 0.60 |
|  | Chemotherapy-Niv-Pem-Placebo | 1.45 | 2.53 | (0.00,6.41) | 0.57 |
|  | Niv-Niv+Ipil-Placebo | 1.36 | 2.33 | (0.00,5.92) | 0.56 |
|  | Ipil-Pem-Placebo | 0.72 | 1.16 | (0.00,2.99) | 0.53 |
|  | Ipil-Niv-Niv+Ipil | 0.38 | 1.83 | (0.00,3.97) | 0.84 |
|  | Ipil-Niv+Ipil-Placebo | 0.10 | 2.05 | (0.00,4.12) | 0.96 |
| Grade 3-5 IRP | Ipil-Niv+Ipil-Placebo | 2.40 | 2.86 | (0.00,8.01) | 0.40 |
|  | Chemotherapy-Ipil-Niv-Pem | 2.14 | 2.94 | (0.00,7.91) | 0.47 |
|  | Ipil-Niv-Placebo | 2.02 | 2.57 | (0.00,7.05) | 0.43 |
|  | Niv-Niv+Ipil-Placebo | 0.99 | 3.00 | (0.00,6.86) | 0.74 |
|  | Ipil-Niv-Niv+Ipil | 0.20 | 1.86 | (0.00,3.84) | 0.91 |
|  | Ipil-Pem-Placebo | 0.12 | 3.23 | (0.00,6.46) | 0.97 |

**Table S7 Results of Node-splitting analysis for the assessment of inconsistency for six treatment groups**

| Comparator | Intervention | Direct | | Indirect | | Difference | | P | Tau |
| --- | --- | --- | --- | --- | --- | --- | --- | --- | --- |
|  |  | Coef. | Std. Err. | Coef. | Std. Err. | Coef. | Std. Err. |  |  |
| **Grade 1-5 IRP** | | | | | | | | | |
| Chemotherapy | Niv | 1.86 | 1.05 | 1.57 | 1.69 | 0.29 | 1.99 | 0.88 | 0.00 |
| Chemotherapy | Pem | 2.24 | 1.45 | 2.53 | 1.36 | -0.29 | 1.99 | 0.88 | 0.00 |
| Ipil | Niv | 0.14 | 0.59 | -0.66 | 1.00 | 0.80 | 1.16 | 0.49 | 0.00 |
| Ipil | Niv+Ipil | 1.42 | 0.42 | 2.29 | 1.19 | -0.87 | 1.25 | 0.49 | 0.00 |
| Ipil | Pem | 0.62 | 0.79 | 0.36 | 1.27 | 0.26 | 1.49 | 0.86 | 0.00 |
| Niv | Niv+Ipil | 1.68 | 0.43 | 0.27 | 1.39 | 1.41 | 1.43 | 0.33 | 0.00 |
| Niv | Placebo | 0.09 | 2.01 | -1.46 | 0.99 | 1.55 | 2.24 | 0.49 | 0.00 |
| Niv+Ipil | Placebo | -3.37 | 1.36 | -2.41 | 0.99 | -0.96 | 1.60 | 0.55 | 0.00 |
| Pem | Placebo | -1.75 | 0.63 | -1.89 | 1.67 | 0.14 | 1.79 | 0.94 | 0.00 |
| **Grade 3-5 IRP** | | | | | | | | | |
| Chemotherapy | Niv | -0.48 | 1.42 | 0.76 | 1.94 | -1.25 | 2.41 | 0.60 | 0.00 |
| Chemotherapy | Pem | 1.68 | 1.48 | 0.43 | 1.90 | 1.25 | 2.41 | 0.60 | 0.00 |
| Ipil | Niv | 0.47 | 1.07 | -0.83 | 1.34 | 1.30 | 1.71 | 0.45 | 0.00 |
| Ipil | Niv+Ipil | 0.79 | 0.81 | 0.22 | 1.67 | 0.56 | 1.86 | 0.76 | 0.00 |
| Ipil | Pem | 0.62 | 1.12 | 2.65 | 1.73 | -2.03 | 2.06 | 0.32 | 0.00 |
| Niv | Niv+Ipil | 0.86 | 0.85 | 0.07 | 1.77 | 0.80 | 1.95 | 0.68 | 0.00 |
| Niv | Placebo | 0.09 | 2.01 | -0.70 | 1.77 | 0.79 | 2.68 | 0.77 | 0.00 |
| Niv+Ipil | Placebo | 0.07 | 2.01 | -1.96 | 1.77 | 2.04 | 2.68 | 0.45 | 0.00 |
| Pem | Placebo | -2.19 | 1.49 | -0.49 | 2.07 | -1.71 | 2.55 | 0.50 | 0.00 |

**Figure S1 Risk of bias for included studies**

1. Risk of bias graph: review authors’ judgements about each risk of bias item presented as percentages


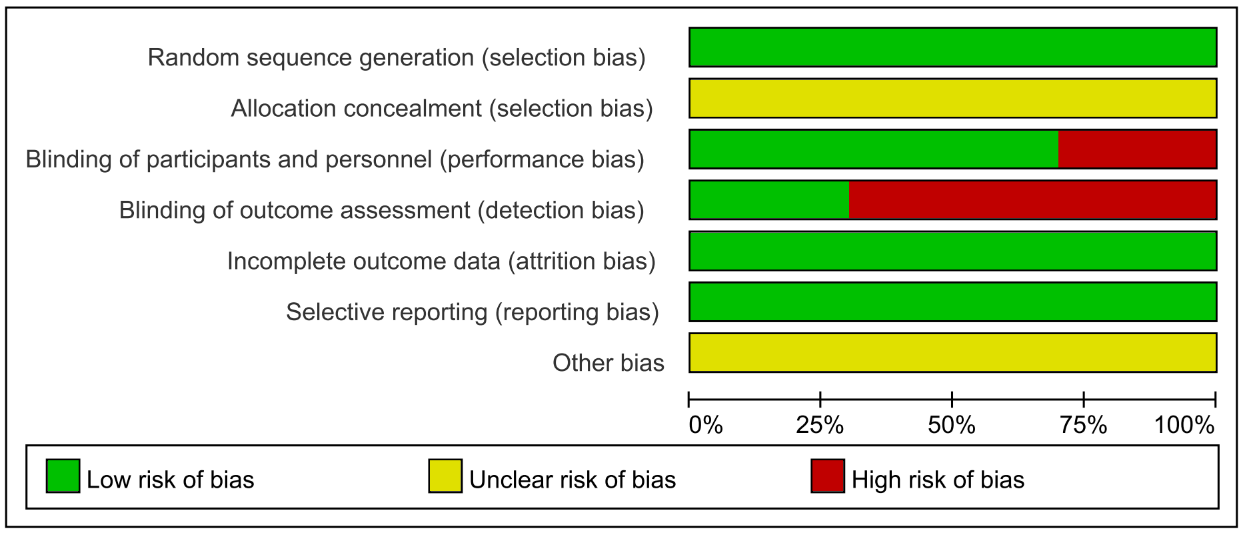


1. Risk of bias summary: review authors’ judgements about each risk of bias item for each included study


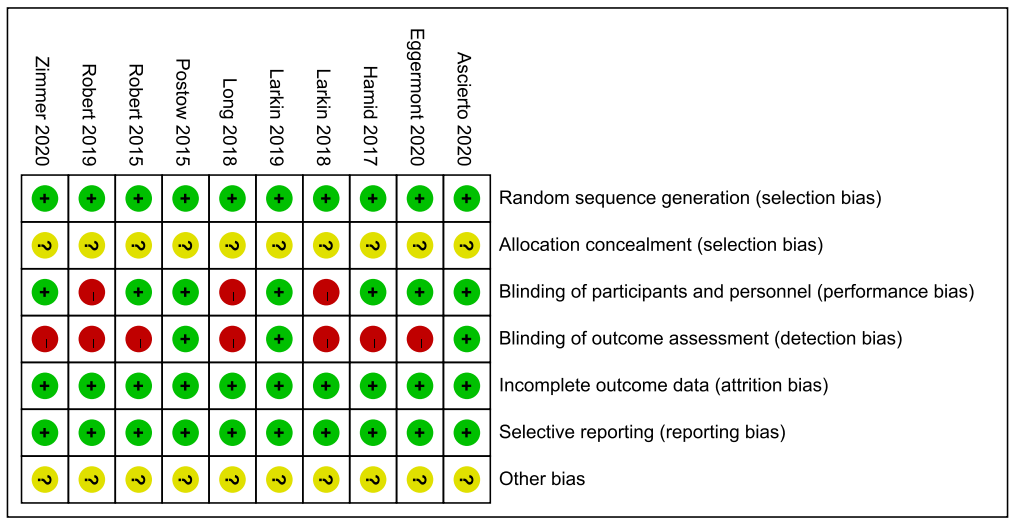


**Figure S2 Publication bias for grade 1-5 IRP in four treatment groups**


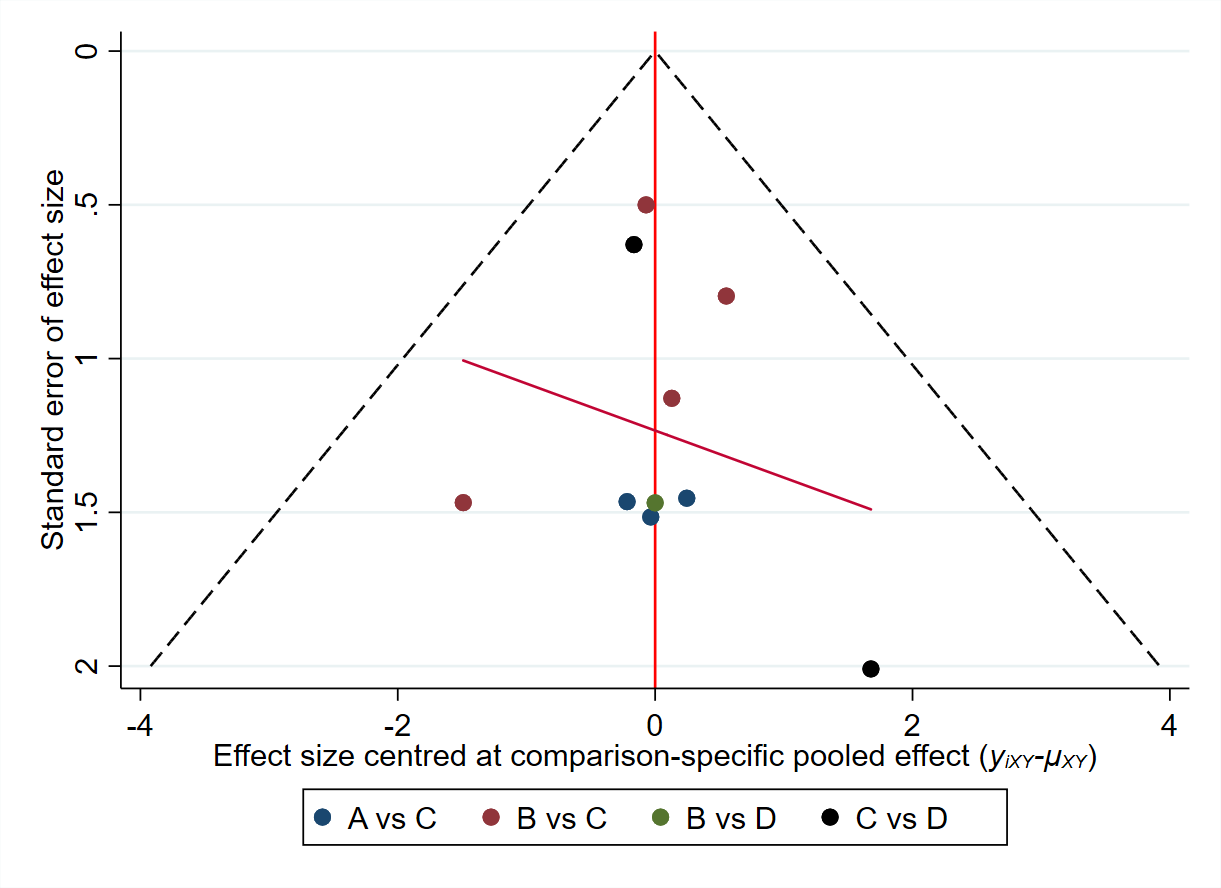


| A：Chemotherapy | B：Dual ICIs combination | C：ICIs monotherapy |
| --- | --- | --- |
| D：Placebo |  |  |

**Figure S3 Publication bias for grade 3-5 IRP in four treatment groups**


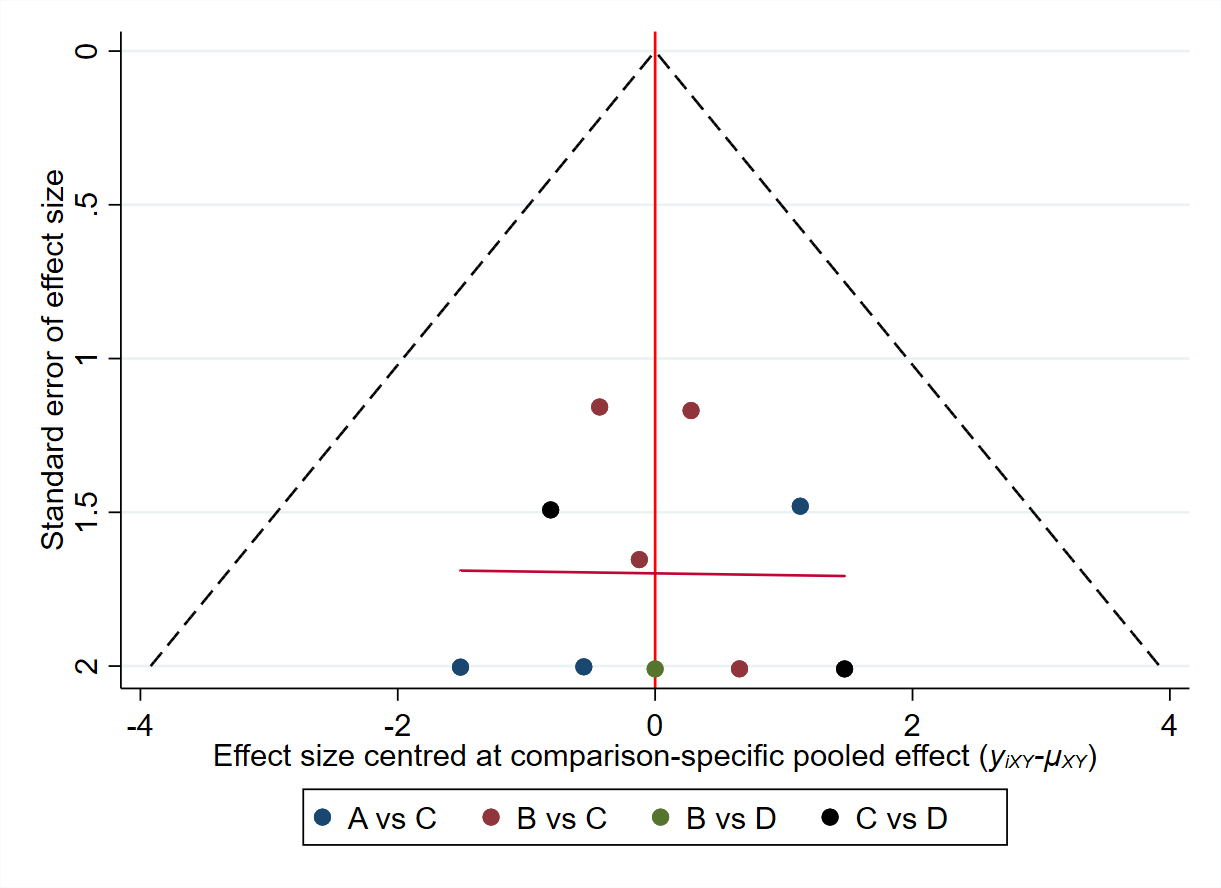


| A：Chemotherapy | B：Dual ICIs combination | C：ICIs monotherapy |
| --- | --- | --- |
| D：Placebo |  |  |

**Figure S4 Publication bias for grade 1-5 IRP in six treatment groups**


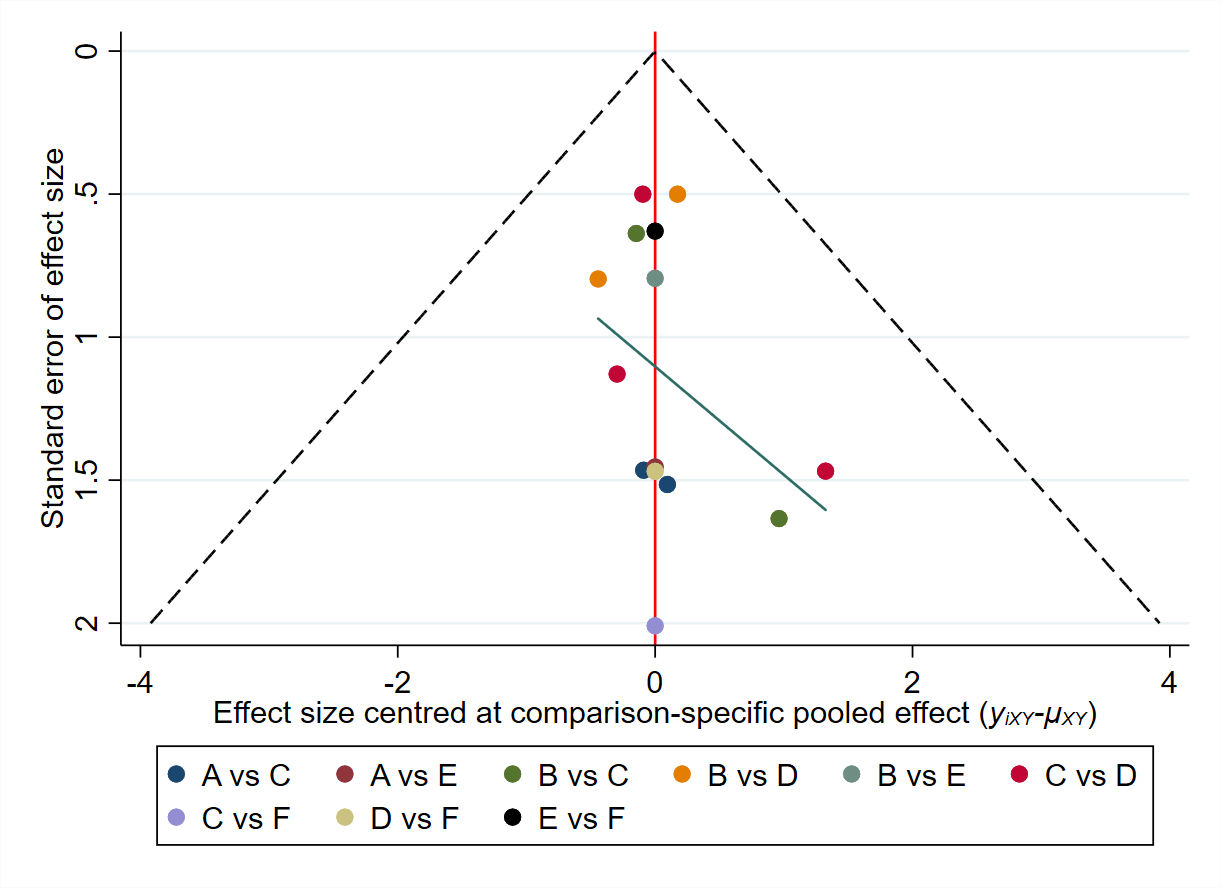


| A: Chemotherapy | B: Ipilimumab | C: Nivolumab | D: Niv + Ipi |
| --- | --- | --- | --- |
| E: Pembrolizumab | F: Placebo |  |  |

**Figure S5 Publication bias for grade 3-5 IRP in six treatment groups**


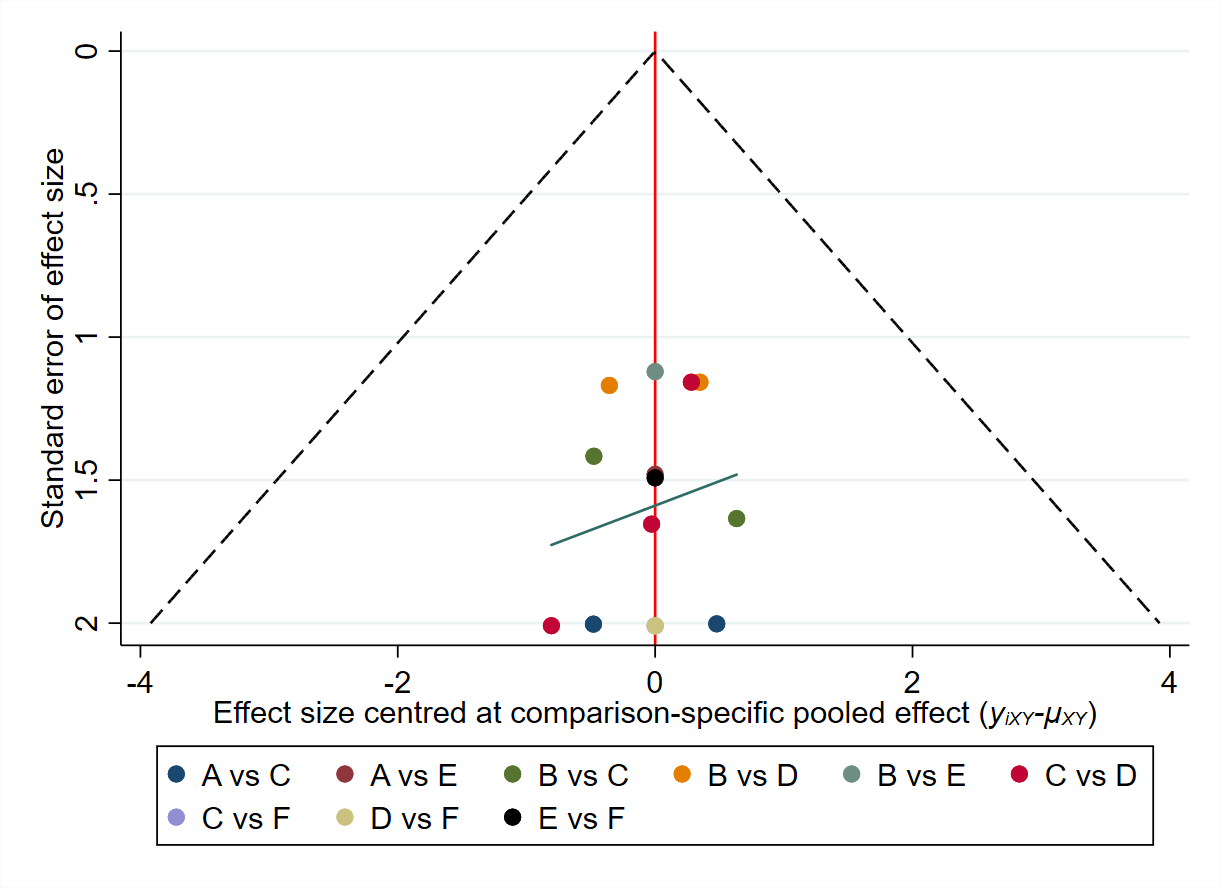


| A: Chemotherapy | B: Ipilimumab | C: Nivolumab | D: Niv + Ipi |
| --- | --- | --- | --- |
| E: Pembrolizumab | F: Placebo |  |  |


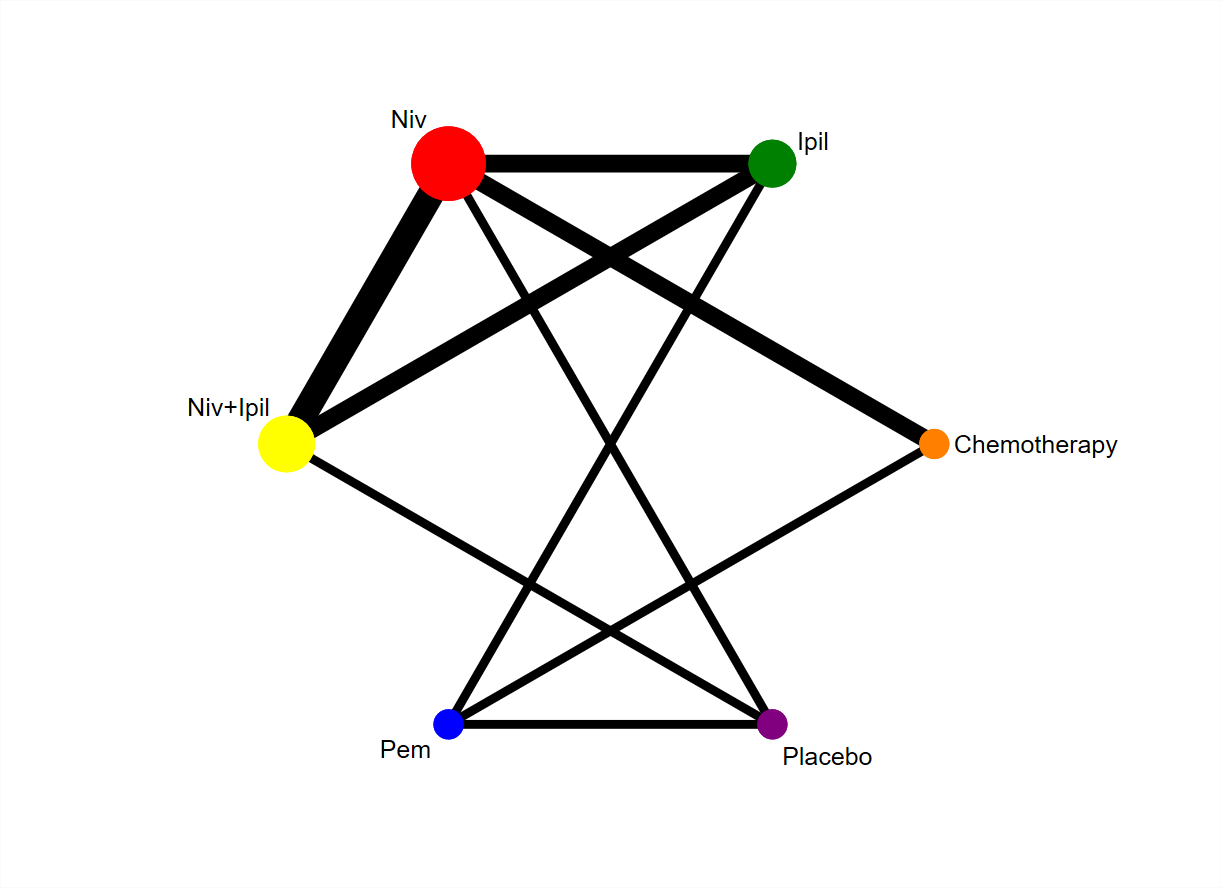


**Figure S6 Network established for comparisons based on six treatment groups. Circular nodes indicate treatment regimens. The node size corresponds with the total number of patients randomized to receive the treatment. Each line represents a type of head-to-head comparison. The width of the lines is proportional to the number of trials comparing the connected treatments.**

*
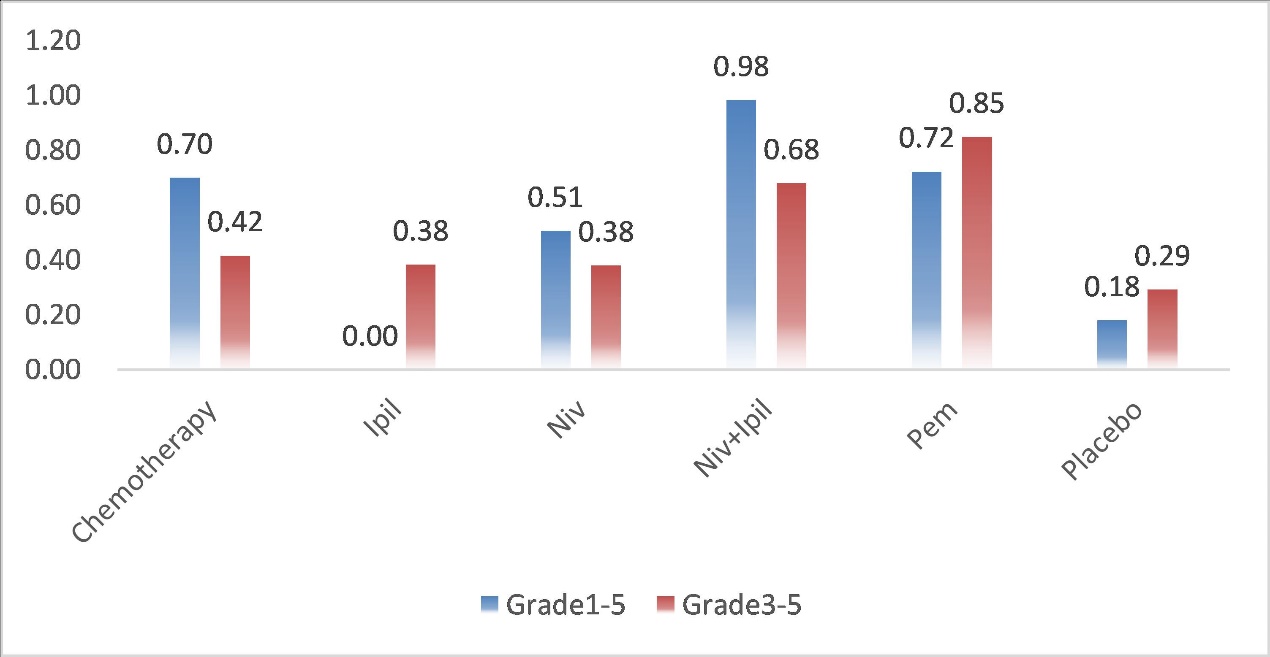
*

**Figure S7 Rank probabilities with SUCRA value for IRP in six treatment groups based on the network consistency model. Higher SUCRA scores are correlated with higher risk of IRP. SUCRA, surface under the cumulative ranking curve.**

**Figure S8 Forest plots and pairwise meta-analysis of head-to-head comparisons for the risk of grade 1-5 IRP in four treatment groups**

**
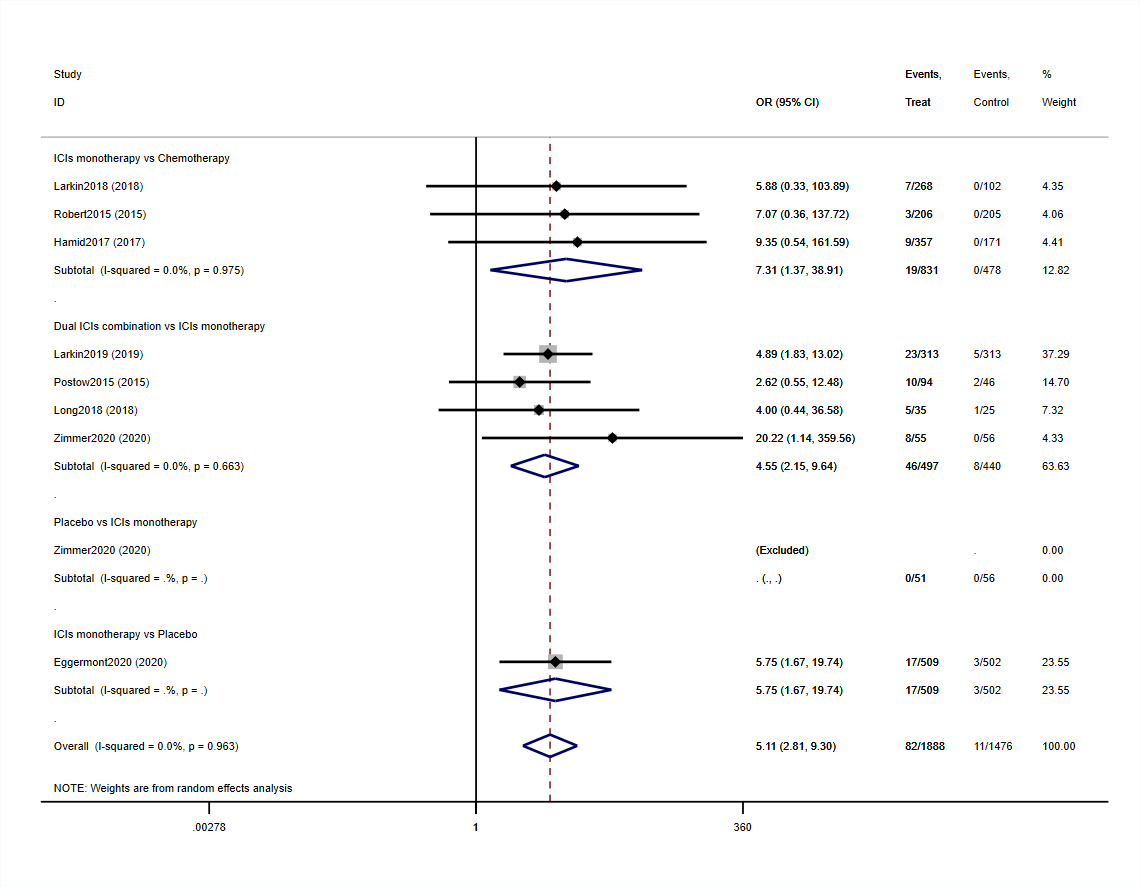
**

**Figure S9 Forest plots and pairwise meta-analysis of head-to-head comparisons for the risk of grade 3-5 IRP in four treatment groups**


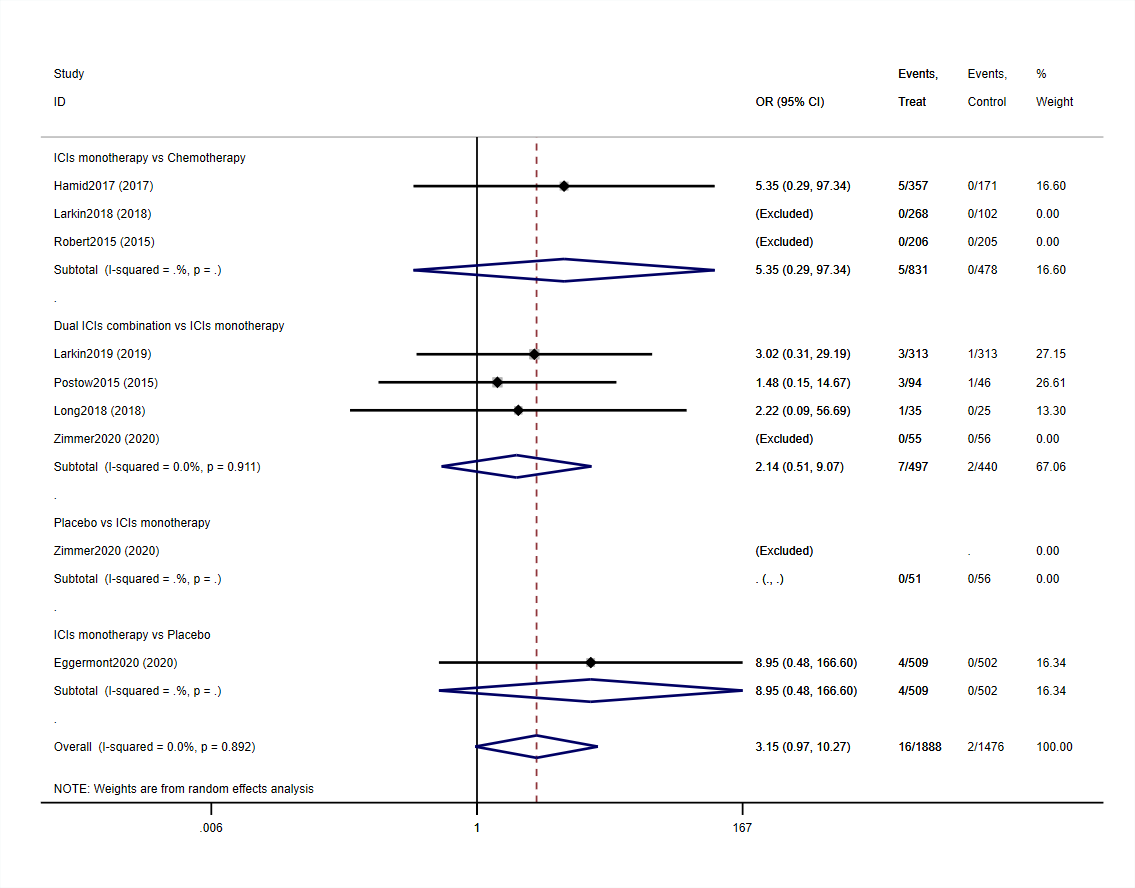


**Figure S10 Forest plots and pairwise meta-analysis of head-to-head comparisons for the risk of grade 1-5 IRP in six treatment groups**


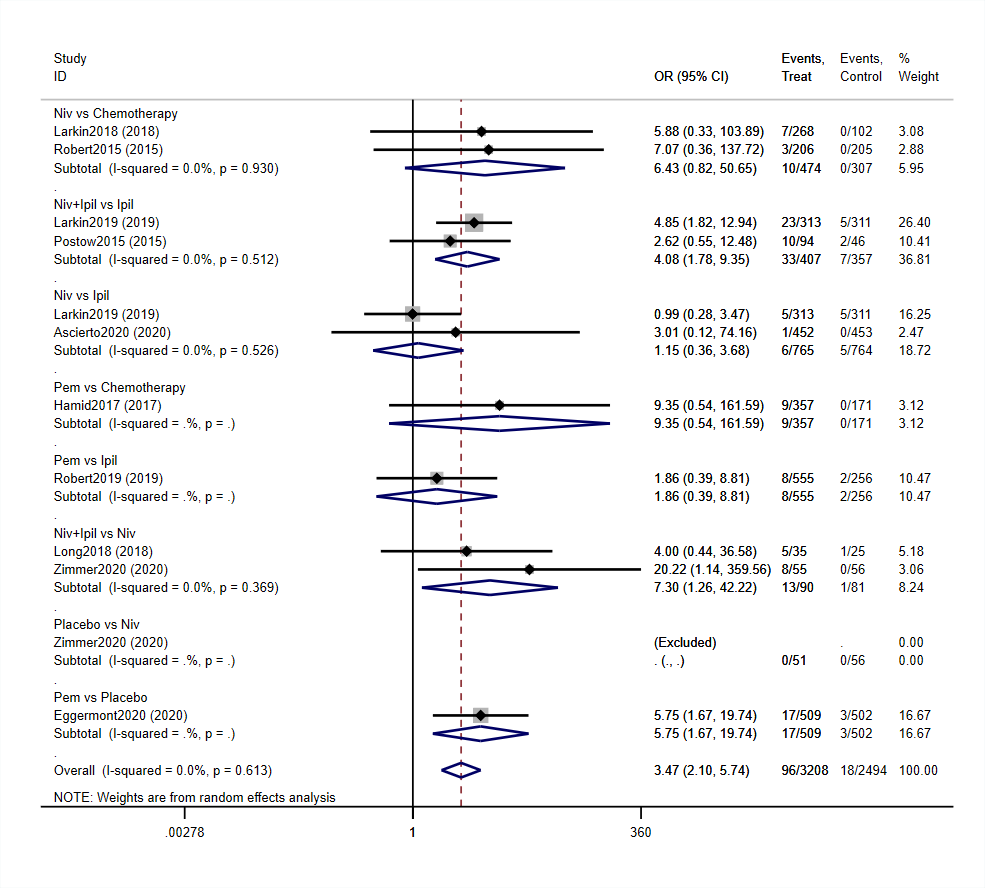


**Figure S11 Forest plots and pairwise meta-analysis of head-to-head comparisons for the risk of grade 3-5 IRP in six treatment groups**


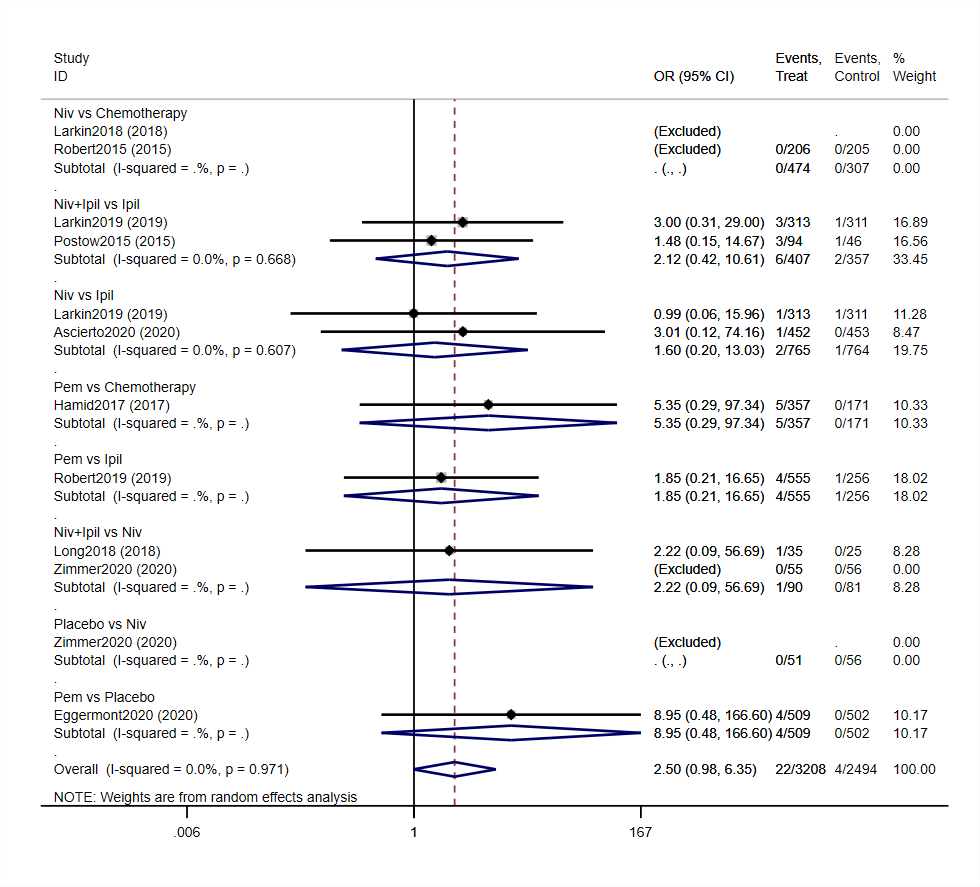

Supplement: Supplementary file 1 [file DataSheet_1.docx]
